# Contrasting Effects of Chitosan Molecular Modifications on the Properties of Fish Gelatin Hydrogels

**DOI:** 10.3390/ijms27209062

**Published:** 2026-10-13

**Authors:** Alena A. Nikiforova, Vladislav Abramov, Polina V. Skvortsova, Aydar A. Akhmadiyarov, Ilnaz T. Rakipov, Andrey V. Sorokin, Maria S. Lavlinskaya, Olga S. Zueva, Marina G. Holyavka, Svetlana R. Derkach, Yuriy F. Zuev

**Affiliations:** 1 Kazan Institute of Biochemistry and Biophysics, FRC Kazan Scientific Center of RAS, Lobachevsky Str. 2/31, 420111 Kazan, Russia; skvpolina@gmail.com (P.V.S.); yufzuev@mail.ru (Y.F.Z.); 2 A.M. Butlerov Chemical Institute, Kazan Federal University, Lobachevsky Str. 1/29, 420008 Kazan, Russia; aydaru@ya.ru (A.A.A.); ilnaz0805@gmail.com (I.T.R.); 3 Biophysics and Biotechnology Department, Voronezh State University, 1 Universitetskaya Square, 394018 Voronezh, Russia; andrew.v.sorokin@gmail.com (A.V.S.); maria.lavlinskaya@gmail.com (M.S.L.); holyavka@rambler.ru (M.G.H.); 4 Institute of Electric Power Engineering and Electronics, Kazan State Power Engineering University, Krasnoselskaya Street 51, 420066 Kazan, Russia; ostefzueva@mail.ru; 5 Institute of Natural Sciences and Technology, Murmansk Arctic University, 183010 Murmansk, Russia; derkachsr@mauniver.ru

**Keywords:** chitosan, chemical modification, fish gelatin, hydrogels, microstructure, rheological properties

## Abstract

The limited water solubility of chitosan, which necessitates acidic conditions for its dissolution, restricts its application in food and pharmaceutical systems. To overcome this limitation, we chemically modified a chitosan molecule by introducing hydrophilic groups and compared three chitosan forms—unmodified chitosan, its quaternary ammonium derivative, and a graft copolymer with poly-*N*-vinylpyrrolidone, with respect to their effect on the structural and mechanical properties of fish gelatin hydrogels. An experimental study combining NMR relaxometry and water self-diffusion measurements, scanning electron microscopy, and rheology revealed a distinct supramolecular architecture of gelatin–chitosan hydrogel network for different chitosan modifications. The NMR data gave information on the molecular mobility of water as a marker of hydrogel structure to elucidate molecular basis for the observed rheological properties. Unmodified chitosan maximally reinforces gelatin network and enhances its thermorheological stability. Chitosan grafting with poly-*N*-vinylpyrrolidone produces a fine-pored xerogel structure with higher thermorheological stability, possibly through a non-cooperative reinforcement mechanism. The quaternary ammonium derivative results in a heterogeneous xerogel morphology with weak mechanical strength and low gel–sol transition temperature. These findings establish the structure–property correlation, demonstrating that chitosan modification governs the molecular network architecture and macroscopic performance of gelatin-based hydrogels, providing molecular-level insights into the behavior of fish gelatin–chitosan systems.

## 1. Introduction

Due to their advantageous properties, such as biocompatibility and the ability to form three-dimensional polymer networks capable of holding large amounts of water, biopolymer-based hydrogels are increasingly used in various technologies and industries, including the food industry [1,2,3], cosmetology [4,5], pharmaceuticals [6,7], medicine [8], and tissue engineering [9,10]. Among the many hydrogel systems, gelatin-based gels, derived from a product of partial collagen hydrolysis, occupy a special place [11]. Gelatin derived from the skin and bones of cattle and pigs has traditionally been used in various industries; but, in recent years, there has been a steady trend toward the use of alternative gelatin sources, such as chicken skin [12,13] or fish cartilage and skin [14,15].

Fish gelatin is of particular interest because its production is actively developing through the processing of fish waste, which aligns with the principles of sustainable development and the circular economy. Moreover, the use of fish gelatin helps overcome religious restrictions associated with animal-derived gelatin and reduces risks related to epidemiological factors [16,17,18]. However, compared with mammalian gelatin, fish gelatin has several apparent disadvantages, including reduced gelling ability and lower gelation and melting temperatures [19]. This is due to its specific amino acid composition: fish gelatin contains lower levels of glycine, proline, and hydroxyproline than mammalian gelatin [19], which play a key role in the formation and stabilization of collagen-like triple helices [20,21]. These limitations greatly restrict the practical application of fish gelatin hydrogels in areas requiring high thermorheological stability and mechanical strength of the resulting gels.

One way to improve the functional properties of fish gelatin gels is to form composite systems with polysaccharides. Our previously obtained data indicate that the introduction of polysaccharides (agar [22,23], alginate [24,25,26], κ-carrageenan [24,27], and chitosan [24,28]) markedly affects the structural and mechanical characteristics of fish gelatin gels by forming a denser and more ordered polymer structure through additional intermolecular interactions. Unlike other polysaccharides, chitosan, as a polycation, causes appreciable changes in hydrogel properties mainly through hydrophobic forces and hydrogen bonds rather than electrostatic interactions. In acidic media, a synergistic effect on the rheological properties of the resulting hydrogel is observed at a low mass ratio of gelatin to chitosan in the range of 10:0.3 to 10:1.5 [28]. In addition to its effects on the rheological characteristics of resulting gels, chitosan is of interest because of its known biological activity, including antibacterial properties [29,30]. However, the use of chitosan is associated with a serious technological problem: it is insoluble in water at neutral and physiological pH values [31]. Its dissolution requires the use of dilute acids, after which the pH of the solution must be adjusted to the required value. This creates evident difficulties in hydrogel preparation, since variations in acidity and ionic strength can noticeably affect the gelation process and the final properties of the materials.

**Figure 1:**
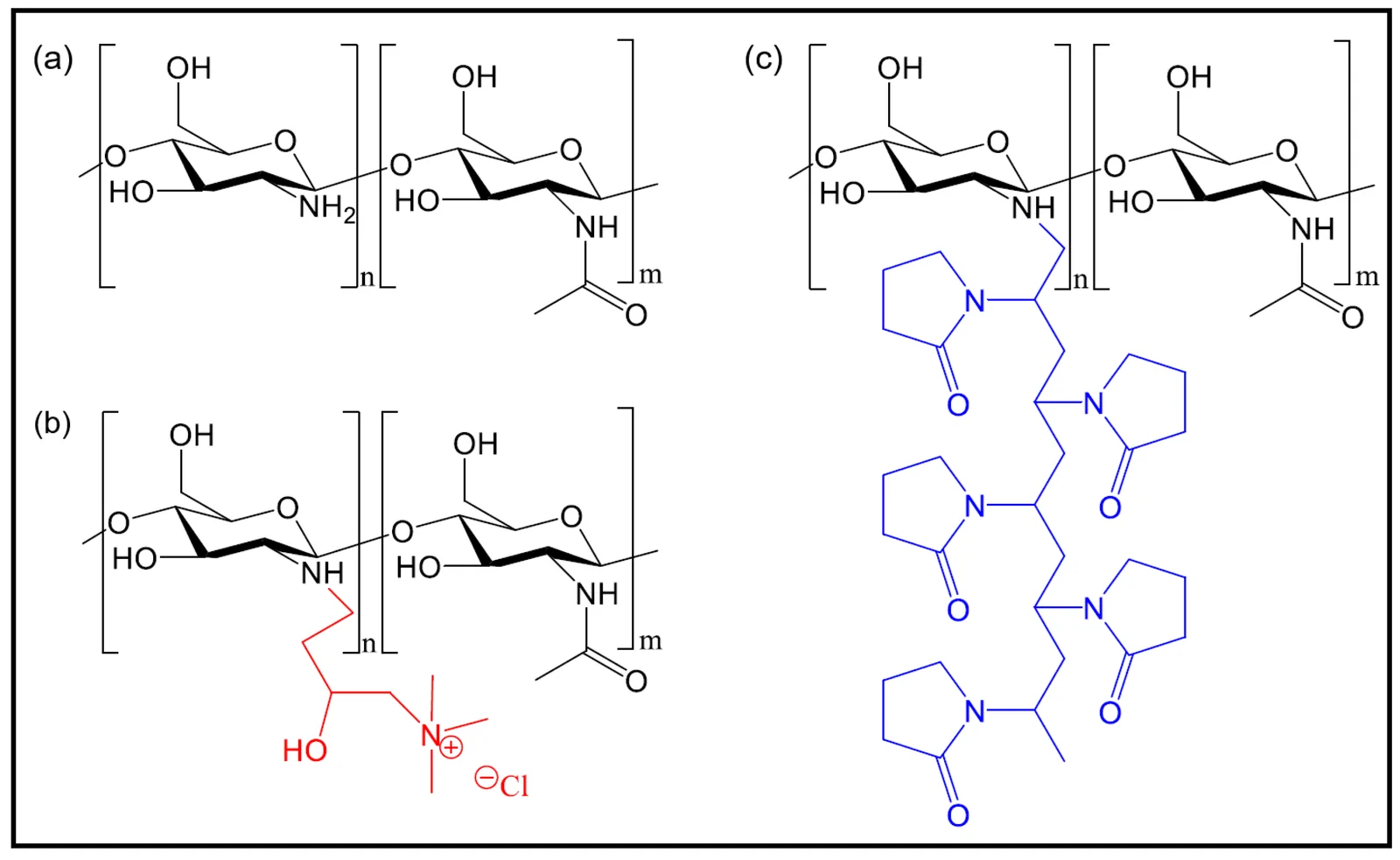
Structures of unmodified chitosan (**a**); *N*-(2-hydroxypropyl)-3-trimethylammonium chitosan (**b**); and the graft copolymer of chitosan with poly-*N*-vinylpyrrolidone (**c**). For clarity, the groups introduced into the base chitosan chain are shown in color.

This limitation can be overcome through chemical modification of chitosan molecular structure (Figure 1a). This approach increases the water solubility while maintaining the biocompatibility and biological activity of the parent polysaccharide [32]. Remarkably, chitosan exhibits the highest susceptibility to chemical modification among known polysaccharides [32]. The *N*-quaternized chitosan has been shown to have excellent water solubility at neutral pH values [31]. Polysaccharide modification is often carried out by graft copolymerization. This method allows one polymer to be attached to another. The process begins with the formation of reactive centers on the original polysaccharide chain, followed by polymerization of the added monomer at these sites, resulting in a graft copolymer [33,34]. In addition to chitosan graft copolymers, considerable attention has recently been paid to the synthesis and investigation of quaternary ammonium derivatives of chitosan [35].

The modified water-soluble chitosan derivatives offer a technological advantage by eliminating the need for acidification and neutralization steps during hydrogel preparation. The introduction of hydrophilic substituents into the chitosan chain may disrupt cooperative interactions with gelatin, thereby affecting the structural and mechanical properties of the composite hydrogel. The present study, therefore, addresses two interconnected aims: (i) a systematic comparison of the effects of two modified chitosan forms—the quaternary ammonium derivative (ChHP, Figure 1b) and the poly-*N*-vinylpyrrolidone (PVP) graft copolymer (ChPVP, Figure 1c)—on the structure and dynamics of fish gelatin (FG) hydrogels relative to unmodified chitosan (Ch, Figure 1a); and (ii) an evaluation of whether these water-soluble modifications can serve as viable substitutes for the native polymer. It should be noted that the antibacterial properties of the modified chitosans, although well documented in the literature, are outside the scope of the present study.

For comprehensive characterization of the obtained hydrogels, a set of physicochemical methods was used. The NMR relaxation and self-diffusion analyses were applied to assess the molecular mobility of water in the gels and to elucidate the molecular basis of the observed rheological data. Scanning electron microscopy was used to analyze the microstructure and morphology of the polymer networks. This multi-scale approach provides molecular-level insight into the relationship between chitosan modification, network architecture, and macroscopic properties, which is essential for the informed design of chitosan-containing hydrogels with predictable performance.

## 2. Results

### 2.1. Water Self-Diffusion

**Figure 2:**
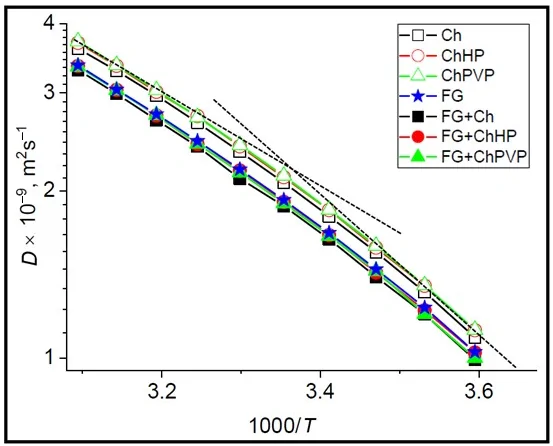
Temperature dependence of the water self-diffusion coefficient in the studied samples. The dashed lines represent linear fits used to calculate the activation energies of water self-diffusion.

The temperature dependence of the water self-diffusion coefficient is an informative parameter for studying spatial confinement in biopolymer hydrogels [36,37]. Figure 2 shows the temperature dependence of the water self-diffusion coefficient, *D*, for all studied systems. The main objective of this experiment was to determine whether our chitosan modifications affected the structure of the polymer network in either chitosan hydrogels or combined gelatin–chitosan hydrogels. The curves are not strictly linear: the temperature dependence of water diffusion in each system exhibits an inflection as a result of the gel–sol phase transition, the temperature of which is difficult to determine precisely because of the small differences in the slopes and the limited temperature interval. Analysis of the experimental curves yielded two main results. The temperature dependence of water self-diffusion is very similar for all studied systems, reflecting the insensitivity of this parameter to individual features of the gel–sol phase transition, even when such differences exist. This can be attributed to the fact that the mesh size of the polymer network in all systems, in both phase states, is large enough that it does not strongly restrict the diffusion of water. The second result is that the presence of gelatin decreases *D* by 8–10%. Evidently, gelatin introduces additional resistance to water diffusion through both mechanical barriers and molecular interactions with the protein surface, which slightly reduce the average translational mobility of water.

The activation energies of water self-diffusion in the gel and sol states were calculated from the slopes of the terminal sections of each curve, shown as dashed straight lines in Figure 2. The obtained activation energies are listed in Table 1.

In the studied systems, the activation energy of water self-diffusion in the sol state is about 18 kJ mol^−1^, which corresponds to the value for pure water [38], indicating that water diffusion is not restricted by the biopolymer network. However, in the gel state, the activation energies are slightly higher, in the range of 21–23 kJ mol^−1^. Evidently, gelation makes the polymer mesh more rigid, affecting the average water mobility. It is worth noting that similar diffusion-related activation energies have been reported for water in curdlan gels [39], water in agarose gels [40], and for lactose and lactic acid in calcium alginate gels [41].

### 2.2. NMR Relaxation

To assess the molecular mobility of water as a structural marker in the studied hydrogels from another side, the longitudinal (spin–lattice, *T*_1_) and transverse (spin–spin, *T*_2_) relaxation times of water protons were measured. Both water proton relaxation times are the population-weighted averages that reflect fast (*T*_1_) and slower (*T*_2_) molecular motion, related to the correlation time of random motion of water molecules, τ_c_, as [36,37,42,43]

(1) *T*_1_, *T*_2_ ∼ 1/τ_c_

The additional contribution to *T*_2_ arises from the magnetization transfer between water and accessible protons of the biopolymer through chemical exchange and/or cross-relaxation. Thus, the *T*_2_ value of water reflects the mobility of biopolymer chains through chemical exchange between water protons and accessible exchangeable protons of the biopolymer.

**Figure 3:**
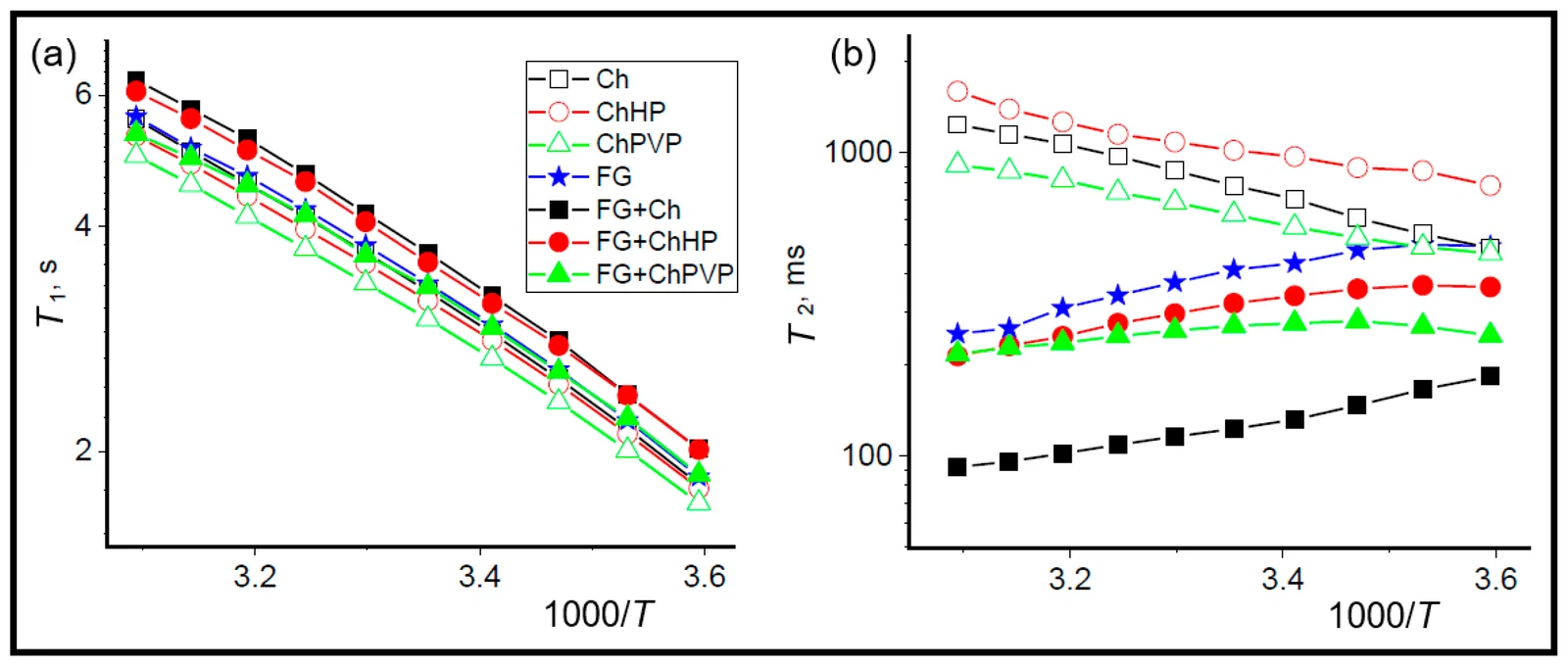
Temperature dependence of water relaxation times *T*_1_ (**a**) and *T*_2_ (**b**) in the studied systems.

Analysis of the *T*_1_ temperature dependences (Figure 3a) revealed no apparent differences in this parameter among the studied systems. A slight decrease in *T*_1_ was observed among the chitosan samples in the order Ch < ChHP < ChPVP. The same trend was observed for the gelatin–chitosan hydrogels compared with the pure gelatin system. This indicates that the modified chitosan forms, especially ChPVP, slightly restrict water mobility within the biopolymer network. This effect may be attributed to the hydrogen-bonding capability of the pyrrolidone carbonyl groups, which could act as the transient binding sites for water. However, the direct evidence for this interaction is not provided by the present data.

In contrast to *T*_1_, which shows the progressive influence of the addition of various chitosan forms on water mobility, the *T*_2_ values in the studied systems are clearly divided into two groups (Figure 3b). For systems without gelatin, the effect on *T*_2_, reflecting a decrease in water mobility, follows the order ChHP > Ch > ChPVP. This indicates that ChPVP forms the most rigid structure among the studied chitosan forms. In addition, the temperature dependence of *T*_2_ follows the same trend as that of *T*_1_ and mainly reflects the intrinsic temperature dependence of water mobility, which is a consequence of the weak interaction of water with chitosan in the studied systems. In contrast, the *T*_2_ behavior of water in the combined gelatin–chitosan systems is markedly different. A notable contribution from chemical exchange and/or cross-relaxation can be seen as a result of water interaction with the extensive gelatin interface [15]. The greatest effect on the averaged properties of the biopolymer surface is exerted by native (unmodified) chitosan, whereas both modified chitosan forms have a smaller effect. In the presence of gelatin, the order of the effect is ChHP < ChPVP < Ch. Thus, in the gelatin-containing systems, the greatest structuring effect, manifested as a decrease in *T*_2_, is caused by unmodified chitosan (Ch). This may indicate that chitosan modifications, while enhancing their own structure-forming properties, can disrupt the cooperativity of gel network formation when combined with gelatin by disturbing the regular arrangement of polysaccharide chains through the irregular distribution of introduced side groups along the polymer backbone.

### 2.3. Microstructure of Xerogels

**Figure 4:**
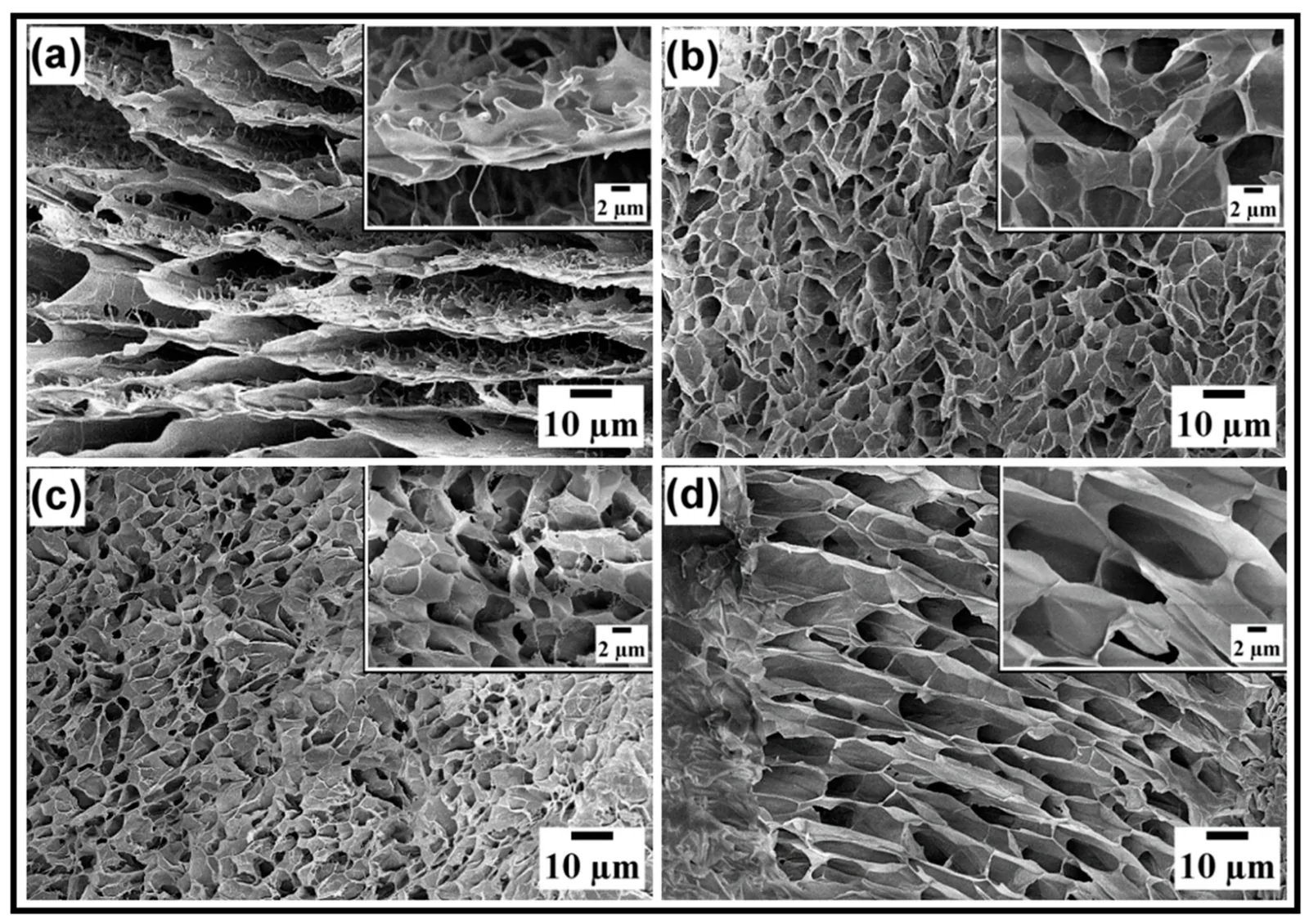
Micrographs of xerogels of 5% fish gelatin (**a**) and gelatin–chitosan combined systems for Ch (**b**), ChPVP (**c**), and ChHP (**d**).

The scanning electron microscopy (SEM) was used to visualize and quantitatively compare the morphology of fish gelatin and gelatin–chitosan xerogels (Figure 4). Since freezing and lyophilization can affect the resulting morphology through the ice formation and drying, the observed xerogel structure should not be considered as a direct representation of the hydrated hydrogel network. Nevertheless, SEM provides a useful approach for the comparative morphological characterization of samples prepared under the same conditions [28,44,45]. Analysis of the micrographs revealed the clear composition-dependent differences in xerogel morphology upon the introduction of chitosan and its modifications.

**Figure 5:**
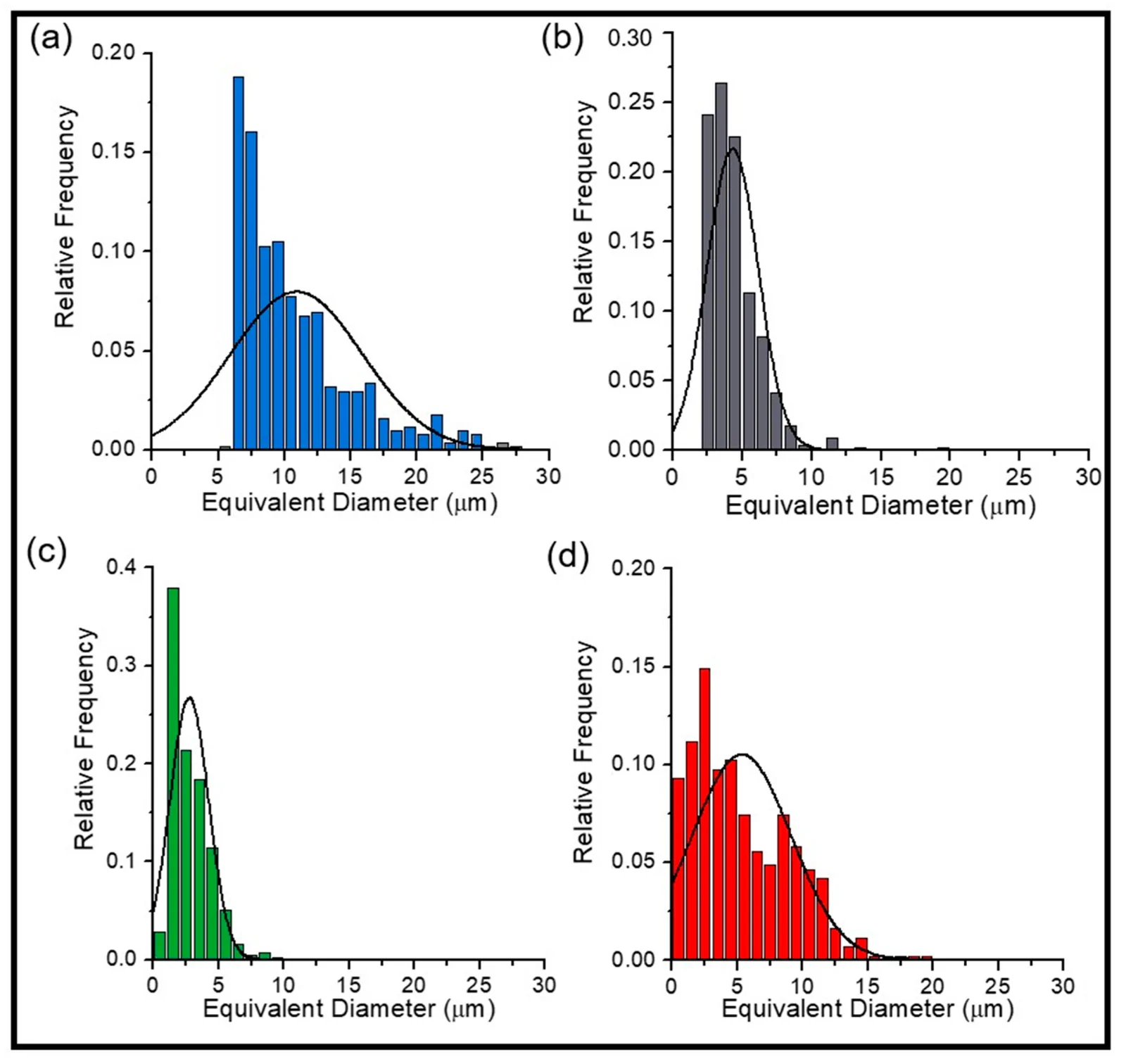
Histograms of cell-size distribution for xerogels fish gelatin (**a**) and gelatin–chitosan for unmodified Ch (**b**), ChPVP (**c**) and ChHP (**d**).

The xerogel based on pure fish gelatin is characterized by an ordered lamellar structure with elongated, oriented pores (Figure 4a). The average pore size, determined from the distribution histograms (Figure 5), was 10.9 ± 5.0 µm, indicating a fairly homogeneous structure with relatively large cells. The introduction of unmodified shrimp chitosan (Ch) leads to a change in the xerogel microstructure (Figure 4b): a transition to a cellular morphology with polygonal pores and massive walls is observed. This indicates the active participation of chitosan in the polymer network formation [43,45]. The average pore size for the FG/Ch sample is 4.3 ± 1.8 µm, which is about 2.5 times smaller than that of pure gelatin. The smaller pore size observed for the FG/Ch xerogel correlates with the NMR relaxometry data, in which Ch causes the maximum shortening of the transverse relaxation time *T*_2_, indicating the formation of the most rigid network.

The chemical modification of chitosan exerts a pronounced but multidirectional influence on the microstructure of the composites. The introduction of ChPVP promotes the formation of a more regular and ordered network structure (Figure 4c). In this case, the average pore size decreases as for unmodified chitosan, amounting to 2.8 ± 1.5 µm, the smallest value among all studied samples. Moreover, the pore size distribution becomes narrower and more uniform, which is consistent with the visually observed ordering. Such structuring is probably due to the ability of the carbonyl groups of the pyrrolidone moieties to form additional hydrogen bonds with the peptide groups of gelatin, which could provide additional physical crosslinking. The use of ChHP leads to the formation of a heterogeneous structure with elongated pores of various sizes (Figure 4d). The average pore size is 5.3 ± 3.8 µm, which is slightly larger than that for unmodified chitosan. The broad pore size distribution is consistent with the visually observed heterogeneous morphology. The steric hindrance created by the bulky trimethylammonium groups apparently disrupts the efficient packing of polymer chains, which may contribute to the more heterogeneous xerogel morphology. This observation is also consistent with the relatively weak effect of ChHP on *T*_2_ in the hydrated system.

### 2.4. Rheological Properties

To quantitatively evaluate the viscoelastic properties and thermorheological stability of hydrogels, the rheological measurements were performed under oscillatory shear. The obtained data allowed correlations to be established between the form of chitosan modification and mechanical characteristics of gelatin-based hydrogels.

**Figure 6:**
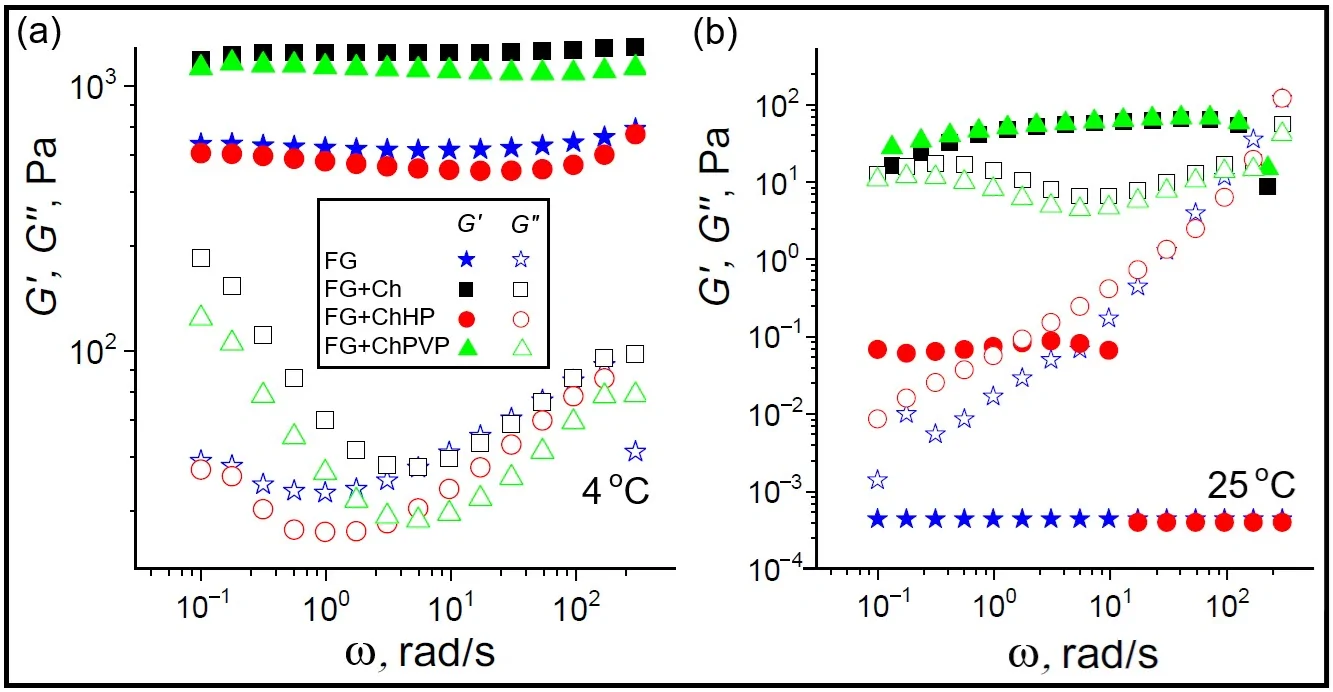
Frequency dependences of storage modulus *G*′ (closed symbols) and the loss modulus *G*″ (open symbols) at 4 °C (**a**) and 25 °C (**b**) for 5% FG, and combined gelatin–chitosan (5% + 0.5%) hydrogels.

Frequency dependences of the storage modulus (*G*′) and loss modulus (*G*″) at 4 °C and 25 °C are presented in Figure 6. At 4 °C, for all studied samples, *G*′ considerably exceeds *G*″ over the entire frequency range (0.1–300 rad/s), indicating the formation of a stable gel network with a predominantly elastic response. The pure fish gelatin gel, at a frequency of 1 rad/s, exhibits a storage modulus of *G*′ = 586 Pa. The addition of unmodified chitosan leads to an approximately 2.3-fold increase in *G*′ (up to 1340 Pa), which is consistent with the literature data [46,47], where an increase in chitosan concentration results in an exponential growth of elastic modulus. This reinforcement is due to the formation of intermolecular interactions between gelatin and chitosan, which creates additional crosslinking junctions in the polymer network [28]. The addition of ChPVP also provides a twofold increase in *G*′ to 1180 Pa. The enhancement of elastic properties in this case may be attributed to the ability of the carbonyl groups of the pyrrolidone moieties to form hydrogen bonds [48] with peptide groups of gelatin, working as additional physical crosslinking agents. In contrast, the addition of ChHP does not lead to reinforcement of the hydrogel: the *G*′ value is 520 Pa, which is somewhat lower than that of the fish gelatin hydrogel without additives.

At 25 °C, the pure gelatin hydrogel completely transforms to the sol state (*G*′ ≈ 0) due to disruption of the collagen-like triple-helical structure of gelatin at temperatures above ambient. The gelatin/ChHP system also exhibits low modulus values; however, in the low-frequency region (below 1.5 rad/s), *G*′ slightly exceeds *G*″, indicating the preservation of a weak gel structure. In contrast, the gelatin systems with unmodified chitosan or ChPVP retain gel-like behavior at 25 °C. At 1 rad/s, the *G*′ values for the gelatin systems with unmodified chitosan and ChPVP are 45 Pa and 48 Pa, respectively, which are approximately 25–30 times lower than at 4 °C, but still sufficient to maintain the structural integrity of the hydrogel. The moduli crossover points, if reliable, occur in the high-frequency region (212 and 232 rad/s for unmodified chitosan and ChPVP, respectively), suggesting the presence of a weak but persistent network.

**Figure 7:**
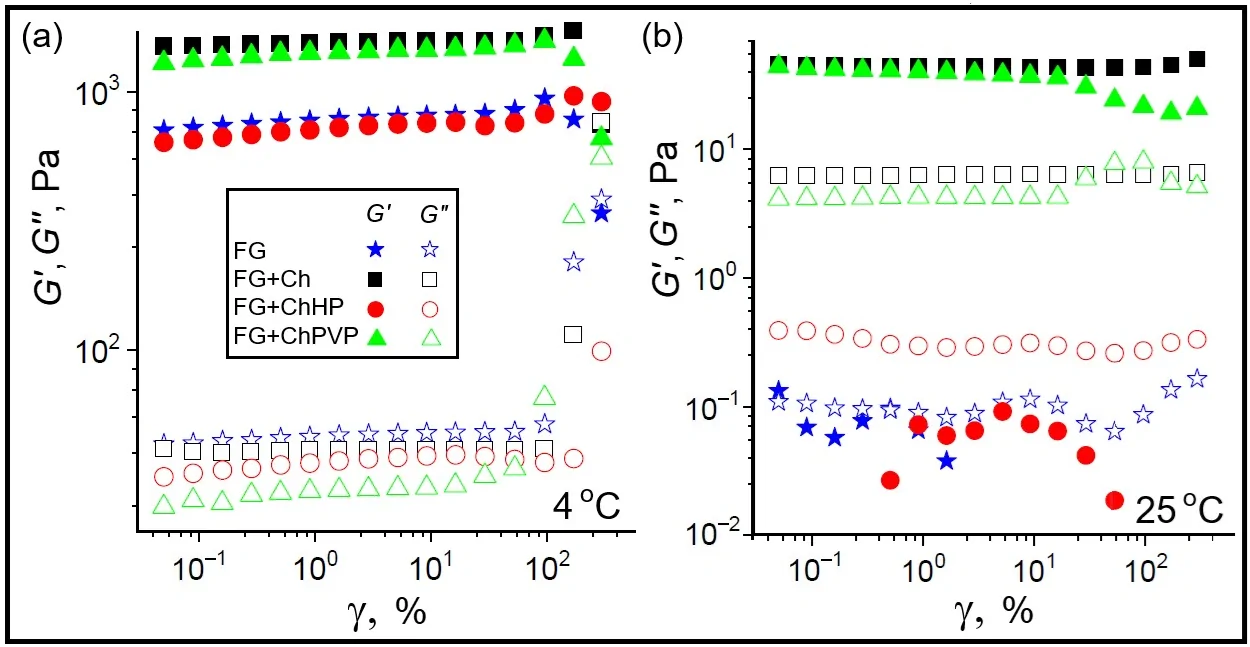
Amplitude dependences of storage modulus *G*′ (closed symbols) and the loss modulus *G*″ (open symbols) at 4 °C (**a**) and 25 °C (**b**) for 5% FG, and combined gelatin–chitosan (5% + 0.5%) hydrogels.

Amplitude sweeps at 4 °C (Figure 7a) revealed an extended linear viscoelastic region (LVR) for all samples, which is a characteristic of stable gel systems [49]. Pure gelatin gel exhibited a storage modulus of about 774 Pa. The addition of unmodified chitosan increases *G*′ approximately twofold (to 1550 Pa). The sample with ChPVP shows *G*′ ≈ 1410 Pa. The gelatin–ChHP hydrogel displays a storage modulus comparable to that of pure gelatin (about 711 Pa). The crossover of *G*′ and *G*″ for pure gelatin and the gelatin-unmodified chitosan sample occurs at strains of about 230–290%, indicating high resistance of the gel networks to mechanical disruption. At 25 °C (Figure 7b), pure gelatin and the gelatin–ChHP system are in the sol state. The gelatin-unmodified chitosan sample maintains the stable *G*′ values of about 44 Pa throughout the entire strain range studied, with no signs of structural breakdown. The gelatin–ChPVP hydrogel gives a comparable storage modulus (about 40 Pa) at small strains; however, starting from a strain of about 16%, a gradual decrease in *G*′ accompanied by an increase in the loss modulus is observed, indicating partial disruption of the polymer network. This difference in mechanical stability may be attributed to the fact that local electrostatic contacts between the carboxylate groups of gelatin and the protonated amino groups of chitosan are more resistant to deformation than the linkages through the grafted poly-*N*-vinylpyrrolidone chains.

**Figure 8:**
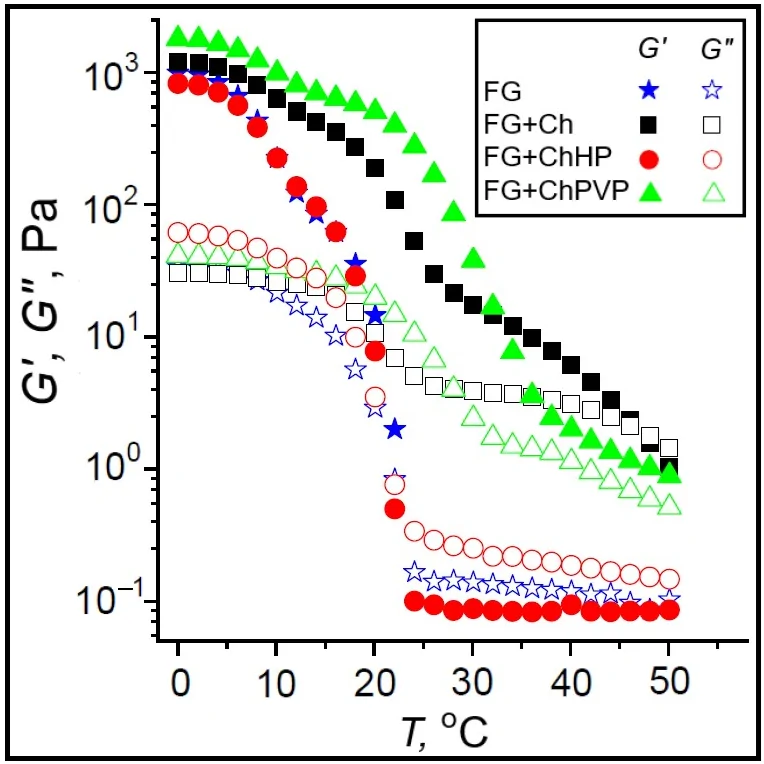
Temperature dependences of storage modulus *G*′ (closed symbols) and the loss modulus *G*″ (open symbols) for 5% FG, and combined gelatin–chitosan (5% + 0.5%) hydrogels.

The temperature dependences of *G*′ and *G*″ (Figure 8) allowed the gel–sol transition temperature (*T*_g–s_) to be determined as the crossover point of the moduli. For the pure fish gelatin hydrogel, it is equal to 22.9 °C, above which the storage modulus is no longer detectable. The introduction of ChHP gives practically no effect on the transition temperature (21.9 °C), which correlates with the frequency and amplitude sweep results showing no strengthening of the gel network. The addition of unmodified chitosan leads to an appreciable increase in the transition temperature up to 47.0 °C, which is 24 °C above the value for the additive-free hydrogel. For the gelatin/ChPVP system, the crossover point of the moduli is absent throughout the entire temperature range studied (up to 50 °C), indicating that the gel network remains thermorheologically stable within this temperature interval. The high thermorheological stability of the ChPVP-containing hydrogels may be attributed to multiple hydrogen bonds formed by the carbonyl groups of pyrrolidone moieties.

## 3. Discussion

Three chitosan forms investigated in this work, the unmodified shrimp chitosan (Ch), its quaternary ammonium derivative (ChHP), and its graft copolymer with poly-*N*-vinylpyrrolidone (ChPVP), differ not only in the chemical nature of the introduced groups but also in the source, molecular weight (MW), and degree of deacetylation (DD) of initial chitosan. These differences raise the question of whether the observed effects on the fish gelatin network can be attributed to the chemical modification itself or to the intrinsic variability of starting materials. A careful analysis of our data and the literature on chitosan-based hydrogels indicates that the primary determinant of their network architecture and macroscopic properties is the nature of the functional groups introduced, rather than the MW or DD of initial chitosan.

The MW and DD of chitosan are known to influence hydrogel properties, particularly the viscosity of solution, the rate of gelation, and the mechanical strength of the resulting network [50]. However, these effects are typically monotonic and relatively modest within the range of MW and DD values used in the present study. If the observed differences in the network rigidity and thermorheological stability were dominated by MW or DD, one would expect ChHP to produce the densest network and the highest storage modulus. In fact, the opposite effect is observed: the ChHP concentration used in the fish gelatin hydrogels yields the loosest network, with *G*′ and *T*_g–s_ values that do not exceed those of pure fish gelatin. Conversely, ChPVP produces the finest and most ordered pore structure and exceptional thermorheological stability. This inversion of the expected trend strongly suggests that the decisive factor is not the intrinsic properties of the initial chitosan but the chemical nature of the substituents introduced.

The degree of substitution (DS) of the modified chitosans also warrants consideration. For ChHP, the DS was 2, meaning that, on average, approximately two quaternary ammonium groups were introduced per anhydroglucosamine unit. This high DS was necessary to achieve water solubility at neutral pH, as the introduction of bulky, hydrophilic groups disrupts the crystallinity of chitosan and facilitates hydration [51,52]. For ChPVP, the PVP content and the grafting frequency reflect a different strategy: the flexible PVP chains provide water solubility through multiple weak hydrogen-bonding interactions with water, without completely consuming the amino groups of the chitosan backbone. In both cases, the DS was chosen to achieve the primary technological goal: water solubility—rather than to optimize network reinforcement. A lower DS might, in principle, preserve more free amino groups for cooperative hydrogen bonding with gelatin, potentially leading to a stronger network. However, such materials would likely be poorly soluble at neutral pH, defeating the purpose of modification. The trade-off between solubility and network-forming ability is therefore intrinsic to the design of water-soluble chitosan derivatives, and the DS values used here represent a reasonable compromise for the intended application.

The molecular-level picture that emerges from the combined NMR, SEM, and rheological data is internally consistent and provides a coherent explanation for the contrasting effects of three chitosan forms. In the gelatin-containing systems, the transverse relaxation time (*T*_2_) of water protons follows the order ChHP > ChPVP > Ch, indicating that the mobility of water is most restricted in the presence of unmodified chitosan and least restricted in the presence of ChHP. This trend is quantitatively mirrored by the average pore size determined from SEM micrographs. A smaller pore size implies a greater fraction of water molecules in close proximity to the polymer surface, where their motion is hindered by hydrogen bonding and chemical exchange with polymer-bound protons. This is consistent with the established mechanism by which *T*_2_ shortening is linked to the network density [53]. The rheological data completes this picture: the storage modulus *G*′ and the gel–sol transition temperature *T*_g–s_ are highest for the Ch-containing system, intermediate for ChPVP, and lowest for ChHP. Thus, all three experimental approaches used probe the same underlying property—the density and connectivity of the polymer network at three different length scales: molecular (NMR), mesoscopic (SEM), and macroscopic (rheology). The consistent trends observed across these different spatial scales provide cross-validation of our conclusions and demonstrate the methodological value of the multi-scale approach applied.

The molecular origin of these differences probably lies in the interaction mechanisms between each chitosan form and gelatin matrix. Unmodified chitosan, despite its poor water-solubility, forms cooperative hydrogen-bonding contacts and local ion-pairs with gelatin, creating a dense and mechanically robust network. This is consistent with the ability of chitosan to form polyelectrolyte complexes with gelatin at pH values near the protein isoelectric point [47]. The ChPVP operates through a different mechanism: the flexible PVP grafts present multiple carbonyl groups that can form hydrogen bonds with the peptide groups of gelatin, acting as additional physical crosslinking agents. Because these interactions are weak and non-cooperative, the resulting network is less rigid than that formed by unmodified chitosan, but it is highly ordered and thermorheologically stable, as evidenced by the absence of a gel–sol transition up to 50 °C. The ChHP, in contrast, introduces bulky quaternary ammonium groups that are permanently positively charged. At the pH of mixed systems, the high charge density of ChHP leads to strong hydration and electrostatic repulsion between polymer chains, preventing the formation of cooperative junctions with gelatin. The steric hindrance of trimethylammonium groups further disrupts the packing of chitosan chains, resulting in a heterogeneous and loose network. This interpretation is consistent with the observed SEM morphology (larger, more heterogeneous pores) and with the lack of reinforcement in the rheological data. It is also consistent with the known behavior of quaternized chitosan in physically blended systems, where the balance between electrostatic attraction and steric hindrance critically determines whether reinforcement or disruption occurs.

It is important to note that the conclusions of this study are specific to the fish gelatin–chitosan system and to the concentration ratio (10:1 mass ratio) used here. The extrapolation of our conclusions to other protein–polysaccharide systems would require the verification of several prerequisites: similarity of the biopolymer pair in terms of charge density and hydrogen-bonding capacity, comparable molecular characteristics of modified polysaccharide, and a similar concentration regime.

## 4. Materials and Methods

### 4.1. Chemicals and Materials

The laboratory-prepared fish gelatin (FG) was extracted from Atlantic cod skin following a previously described procedure [15]. The average molecular weight of FG was determined to be 153 kDa. Chitosan from shrimp shells (Ch), with a degree of deacetylation ≥75%, was purchased from Sigma-Aldrich (Saint Louis, MO, USA). The *N*-(2-hydroxypropyl)-3-trimethylammonium chitosan (ChHP) and a graft copolymer of chitosan with poly-*N*-vinylpyrrolidone (ChPVP) were synthesized according to the procedures described below.

All samples were prepared using deionized water from an Arium Mini water purification system (Sartorius, Göttingen, Germany). Unmodified chitosan was dissolved in a 0.1 M acetic acid solution prepared from glacial acetic acid (PanReac AppliChem, Darmstadt, Germany, 99.5%).

### 4.2. Synthesis of Chitosan Derivatives

The ChPVP graft copolymer was synthesized from chitosan (Bioprogress, Losino-Petrovsky, Russian Federation, molecular weight 200 kDa, degree of deacetylation 91%) and *N*-vinylpyrrolidone (VP; >99%, Sigma, Saint Louis, MO, USA) with a boiling point of 79–81 °C at 5 mm Hg according to the procedure described in [33]. Briefly, chitosan (Ch; 0.5 g) and 50 mL of a 2% (*w*/*w*) acetic acid solution were placed in a Schlenk flask equipped with a magnetic stirrer and an argon purge line. Once the chitosan had completely dissolved, the calculated amount of VP (Ch:VP molar ratio = 1:5) was added. The resulting mixture was degassed by three freeze–pump–thaw cycles. An initiator mixture consisting of the calculated amounts of potassium persulfate (PPS) and sodium metabisulfite (SMBS; PPS:SMBS molar ratio = 1:1; final PPS concentration 2 mM) was then added against the argon flow, and the reaction was kept at 40 °C for 4 h. After polymerization was complete, the mixture was poured into acetone, and the precipitate was isolated by centrifugation. The crude copolymer, which contained homopolymer, was dried under vacuum, weighed, and purified in a Soxhlet apparatus with ethanol to extract the homopolymer and other impurities. The final product was vacuum-dried to constant weight. The grafted PVP chains were isolated by oxidative degradation and analyzed by gel permeation chromatography, yielding a molecular weight of 6850 Da and a PVP content of 45.1%. The grafting frequency was calculated to be 0.0192 per anhydroglucosamine unit.

*N*-(2-hydroxypropyl)-3-trimethylammonium chitosan (ChHP) was synthesized by alkylation of chitosan with glycidyltrimethylammonium chloride. Three grams of chitosan (Bioprogress, Losino-Petrovsky, Russian Federation, molecular weight 200 kDa, degree of deacetylation 91%) was placed in a thermostated reactor equipped with an overhead stirrer and a reflux condenser, dispersed in 30 mL of water at 85 °C, and then 10.65 mL of glycidyltrimethylammonium chloride (Sigma-Aldrich, Taufkirchen, Germany, >90%) was added in three equal portions at one-hour intervals. Two hours after the addition of the last reagent portion, the solution was precipitated into 500 mL of cold acetone and kept in a refrigerator for 12 h. The liquid was then decanted, the obtained precipitate was dissolved in 100 mL of methanol, and reprecipitated into 300 mL of an acetone/ethanol mixture (4:1 *v*/*v*). The precipitate was collected on a Büchner funnel and dried in a vacuum oven at 45 °C to constant weight. The product yield was 37.55%, and the conversion of the alkylating agent was 21.7%. The degree of substitution of the product, calculated from the ^1^H NMR spectroscopy data, was 2.0 ± 0.3. This value indicates that glycidyltrimethylammonium chloride reacted not only with the primary amino group at C-2 but also with the hydroxyl groups at C-3 and C-6 of the glucosamine unit, giving a maximum theoretical DS of 2 for this reaction.

### 4.3. Preparation of Hydrogels

Portions of fish gelatin and modified chitosans were dissolved in water, whereas the shrimp chitosan was dissolved in a 0.1 M acetic acid solution. All suspensions were stirred on a magnetic stirrer C-mag HS 7 (IKA, Staufen, Germany) at room temperature at a stirring speed of 800 rpm for 5 h. Then, for complete dissolution, the gelatin suspension was placed in an ultrasonic bath Sonorex TK52 (Bandelin, Berlin, Germany) at 50 °C, and the shrimp chitosan suspension was placed at 70 °C for 1 h. After dissolution, the shrimp chitosan solution was adjusted to pH ~6 with a 1 N sodium hydroxide solution (Diaem, Moscow, Russian Federation, ≥99%) using a pH meter PB-11 (Sartorius, Göttingen, Germany). The initial concentrations of the gelatin and chitosan solutions were 10% (*w*/*v*) and 1% (*w*/*v*), respectively. The pH values of the solutions were as follows: fish gelatin, 5.93; shrimp chitosan, 5.98; ChHP, 6.21; and ChPVP, 6.52. The warm gelatin solution was added to each chitosan solution to obtain final concentrations of 5% (*w*/*v*) gelatin and 0.5% (*w*/*v*) chitosan. The mass ratio of 10:1 (gelatin:chitosan) used in the present study was therefore selected because it lies at the beginning of this plateau region [28], where the network structure is fully developed and the rheological properties are near their maximum values, while avoiding unnecessary excess of the polysaccharide component. It should be noted that the comparison was performed at equal total polymer concentration, and that this does not correspond to equal chitosan backbone concentration. The solutions were stirred for 1 h in an ultrasonic bath at 40 °C.

The pH value of the 5% fish gelatin solution was 5.92; those of the fish gelatin/Ch, gelatin/ChHP, and gelatin/ChPVP systems were 5.97, 6.12, and 6.24, respectively. The small differences in final pH are unlikely to lead to the large changes in measured characteristics, given that the electrostatic interactions between fish gelatin and chitosan are known to be weak in this pH range. The fish gelatin/Ch system contains sodium and acetate ions introduced during chitosan dissolution in 0.1 M acetic acid and subsequent neutralization with NaOH, but their concentrations are sufficiently low that the resulting ionic strength has a negligible effect on the polysaccharide–protein interactions.

The hydrogels were obtained by storage of these systems at 4 °C for 20 h. Samples were prepared in triplicate.

### 4.4. NMR Relaxation and Self-Diffusion Measurements

^1^H NMR relaxation and water self-diffusion experiments were conducted using a Bruker AVANCE III 600 MHz spectrometer (Bruker BioSpin GmbH, Rheinstetten, Germany) equipped with a triple-resonance BBO probe, z-gradients, and a BCU-05 temperature control unit. Measurements were performed during heating from 5 to 50 °C in 5 °C steps, with samples equilibrated for 10 min at each set point prior to data acquisition. Self-diffusion coefficients were measured using the standard stimulated-echo pulse sequence with bipolar gradient pulses (*stebpgp1s*). The magnetic field gradient amplitude was varied from 1 to 48 G cm^−1^, with a constant diffusion time Δ = 50 ms and gradient durations δ = 1–2 ms. Water relaxation times *T*_1_ and *T*_2_ were measured using the inversion-recovery (*t1ir*) and Carr–Purcell–Meiboom–Gill (*cpmg*, τ = 1.5 ms) pulse sequences, respectively. Data processing and analysis were performed using TopSpin software (version 3.5.7, Bruker, Rheinstetten, Germany). For NMR experiments, 10% by volume D_2_O was added to the aqueous phase.

### 4.5. Scanning Electron Microscopy

The morphology of freeze-dried gelatin and gelatin-polysaccharide hydrogels (xerogels) was examined by SEM using a field-emission scanning electron microscope, Merlin (Carl Zeiss, Oberkochen, Germany), at an accelerating voltage of 5 kV. The experiments were performed at the Interdisciplinary Center of Analytical Microscopy (Kazan Federal University, Kazan, Russian Federation). The initial hydrogel samples were frozen in liquid nitrogen, freeze-fractured, and lyophilized to obtain xerogels. The xerogel sections were sputter-coated with a gold–palladium mixture (80/20) for SEM imaging. For each sample, 15–20 SEM micrographs were obtained at various magnifications.

For quantitative analysis, five micrographs per sample were selected based on their suitability for reliable cell-size measurements. Micrographs in which individual cell boundaries could not be clearly identified due to section orientation or local fracture features were excluded from the analysis. The distribution of equivalent diameters (d_e_q) of xerogel cells was determined using the Mountains SEM^®^ software package (version 11.0, Digital Surf, Besançon, France). Approximately 430–560 cells were analyzed for each sample. d_e_q was calculated as the diameter of a circle having the same area as the measured cell. The minimum object size for analysis was set to 1 μm^2^ to exclude measurement artifacts.

### 4.6. Rheological Measurements

The rheological properties of fish gelatin hydrogels and hydrogels of fish gelatin with different chitosan forms were studied using an MCR-302 automatic rheometer (Anton Paar GmbH, Graz, Austria) equipped with a parallel plate measuring system PP50-1 (plate diameter 50 mm, gap between plates was 1 mm). The instrument and geometry inertia were calibrated using the manufacturer’s standard procedure, and the raw torque signal was corrected for inertia using the software’s built-in compensation. Hydrogel samples were transferred onto the measuring system at 4 °C, then equilibrated to the measurement temperature (4 or 25 °C) and held there for 15 min prior to measurement. Vacuum oil was applied around the perimeter of the loaded sample (gel or sol) to prevent water evaporation. The following measurement protocols were applied:

(1) Oscillation mode under strain control (γ = 0.05–300%) at a constant frequency of f = 1 Hz (ω = 6.28 rad/s);
(2) Oscillation mode under frequency sweep control (ω = 0.1–300 rad/s) at a constant strain amplitude of γ = 1%;
(3) Oscillation mode under temperature control (range 0–50 °C) at constant strain amplitude γ = 1%, frequency f = 1 Hz, and a heating rate of 1 °C/min.

## 5. Conclusions

In this work, we conducted a systematic comparative study of the influence of three molecular forms of chitosan, unmodified chitosan, its quaternary ammonium derivative, and a graft copolymer with poly-*N*-vinylpyrrolidone, on the supramolecular structure and properties of fish gelatin hydrogels. By integrating the NMR relaxation, water self-diffusion analysis, scanning electron microscopy, and rheological measurements, we established a comprehensive molecular-level picture of the effect of different chitosan forms on the network architecture, water dynamics, and rheological properties of the resulting fish gelatin-based composite hydrogels.

Each chitosan derivative induces a distinct molecular network organization. Unmodified chitosan, despite its poor water solubility, presumably forms cooperative intermolecular contacts with gelatin, leading to the mechanically strongest and most deformation-resistant network. The graft copolymer, while producing the finest-pored xerogel morphology and the highest gel–sol transition temperature, may operate through a non-cooperative mechanism based on multiple weak hydrogen bonds rather than rigid network junctions. In contrast, the behavior of the quaternary ammonium derivative may be associated with steric hindrance from the bulky trimethylammonium groups, which could interfere with effective junctions with the gelatin matrix, resulting in a hydrogel whose mechanical properties do not exceed those of pure gelatin.

A particularly informative finding is that none of the modifications appreciably alters the activation barrier for water self-diffusion, which remains close to that of free water. This indicates that even the most compact networks retain pores large enough to allow relatively unrestricted water self-diffusion. While this may be relevant to the transport phenomena in hydrogels, it should be noted that water self-diffusion is not a direct measure of the transport of larger solutes such as drugs, proteins, or nutrients. Dedicated permeability measurements with model solutes would be required to support such applications.

Collectively, these findings establish a structure–property correlation for the fish gelatin–chitosan system studied here, demonstrating that the mode of chitosan modification governs the network architecture and macroscopic performance of these composite hydrogels. Unmodified chitosan is the preferred choice when high mechanical strength and thermorheological stability are required, while the graft copolymer is optimal when maximum thermal resistance is prioritized, even at the expense of rigidity. The quaternary ammonium derivative (degree of substitution = 2), though water-soluble, is unsuitable for reinforcement (5% fish gelatin, 0.5% ChHP), but may be useful in applications where fast degradation is desired.

While the molecular mechanisms proposed in this work are supported by the combination of NMR, SEM, and rheology data, further confirmation using complementary techniques such as FTIR, calorimetry, or molecular modeling is planned. The multi-technique approach employed here offers a robust framework for correlating chemical modification at the molecular level with macroscopic hydrogel performance, and this methodology can be extended to other polysaccharide–protein systems to facilitate the rational development of biomaterials with predefined functional properties for food, cosmetic, and biomedical uses.

## Figures and Tables

**Table 1:** Activation energies of water self-diffusion in the gel and sol states of the studied systems.

Sample	Ea (gel), kJ·mol^−1^	Ea (sol), kJ·mol^−1^
0.5% Ch	23.57	18.30
0.5% ChHP	-	19.13
0.5% ChPVP	-	19.14
5% FG	22.27	18.19
5% FG + 0.5% Ch	22.48	18.12
5% FG + 0.5% ChHP	21.94	18.32
5% FG + 0.5% ChPVP	22.84	18.25

The uncertainties in the activation energies were estimated from the standard errors of the linear regression fits and do not exceed 5%.

## Data Availability

The original contributions presented in this study are included in the article. Further inquiries can be directed to the corresponding authors.

## Abbreviations

The following abbreviations are used in this manuscript:

FG	Fish gelatin
Ch	Unmodified shrimp chitosan
ChHP	*N*-(2-hydroxypropyl)-3-trimethylammonium chitosan
ChPVP	Graft copolymer of chitosan and poly-*N*-vinylpyrrolidone
